# Association of PON1, P2Y12 and COX1 with Recurrent Ischemic Events in Patients with Extracranial or Intracranial Stenting

**DOI:** 10.1371/journal.pone.0148891

**Published:** 2016-02-12

**Authors:** Xiao-Qing Li, Ning Ma, Xin-Gang Li, Bo Wang, Shu-Sen Sun, Feng Gao, Da-Peng Mo, Li-Gang Song, Xuan Sun, Lian Liu, Xing-Quan Zhao, Yi-Long Wang, Yong-Jun Wang, Zhi-Gang Zhao, Zhong-Rong Miao

**Affiliations:** 1 Department of Interventional Neuroradiology, Beijing Tiantan Hospital, Capital Medical University, National Clinical Research Center for Neurological Diseases, Center of Stroke, Beijing Institute for Brain Disorders, Beijing, China; 2 Department of Neurology, Shaanxi Provincial People’s Hospital, Xi’an, China; 3 Department of Pharmacy, Beijing Tiantan Hospital, Capital Medical University, Beijing, China; 4 College of Pharmacy, Western New England University, Springfield, Massachusetts, United States of America; 5 Department of Neurology, Beijing Tiantan Hospital, Capital Medical University, Beijing, China; University of Münster, GERMANY

## Abstract

**Background and Purpose:**

Short-term combined use of clopidogrel and aspirin improves cerebrovascular outcomes in patients with symptomatic extracranial or intracranial stenosis. Antiplatelet non-responsiveness is related to recurrent ischemic events, but the culprit genetic variants responsible for the non-responsiveness have not been well studied. We aimed to identify the genetic variants associated with poor clinical outcomes.

**Methods:**

Patients with symptomatic extracranial or intracranial stenosis scheduled for stenting and receiving dual antiplatelets (clopidogrel 75 mg and aspirin 100 mg daily) for at least 5 days before intervention were enrolled. Ischemic events including recurrent transient ischemic attack, stroke, myocardial infarction, and vascular-related mortality within 12 months follow-up were recorded. We examined the influence of genetic polymorphisms on treatment outcome in our patients.

**Results:**

A total of 268 patients were enrolled into our study and ischemic events were observed in 39 patients. For rs662 of paraoxonase 1 (PON1), allele C was associated with an increased risk of ischemic events (OR = 1.64, 95%CI = 1.03–2.62, *P* = 0.029). The A-allele carriers of rs2046934 of P2Y12 had a significant association with adverse events (OR = 2.01, 95%CI = 1.10–3.67, *P* = 0.041). The variant T-allele of cyclooxygenase-1 (COX1) rs1330344 significantly increased the risk of recurrent clinical events (OR = 1.85, 95%CI = 1.12–3.03, *P* = 0.017). The other single nucleotide polymorphism (SNP) had no association with ischemic events.

**Conclusions:**

PON1, P2Y12 and COX1 polymorphisms were associated with poorer vascular outcomes. Testing for these polymorphisms may be valuable in the identification of patients at risk for recurrent ischemic events.

## Introduction

Clopidogrel is a prodrug, which requires conversion to an active metabolite by multiple enzymes. The active metabolite acts by inhibiting the ADP receptor P2Y12 on platelet cell membranes [1]. Aspirin is a COX-1 inhibitor, preventing the production of TBXA2, which plays an important role in platelet aggregation. Short- term dual antiplatelet therapy appears to be safe and effective in reducing stroke recurrence and combined vascular events in patients with acute ischemic stroke or TIA as compared with monotherapy [2,3].

Although the clinical benefit of a course of dual antiplatelet therapy for patients undergoing stenting of extracranial or intracranial stenosis is undisputed, inter-individual variability in clopidogrel and aspirin response may account for the poor outcome in some patients even after a successful procedure [4–6]. Patients treated with clopidogrel who demonstrate higher *in vitro* platelet reactivity are at an increased risk of ischemic events [6]. Genetic polymorphism is one of the reasons for clopidogrel and aspirin treatment failure [7,8]. The loss-of-function CYP2C19*2 variant had been shown to be associated with decreased active metabolites and increased adverse clinical outcomes in patients treated with clopidogrel [9,10]. Despite its robust association with poor outcomes of clopidogrel therapy, CYP2C19 loss-of-function alleles do not account for all of the variability. The clinical use of the CYP2C19 genotype as a prediction tool for personalized antiplatelet therapy remains debatable.

Aside from CYP2C19, there are many other related genes in the pathway of aspirin and clopidogrel metabolism, such as ABCB1, PON1, CYP2C9, CYP2C18, CES1, P2Y12, COX1 and UCP2. The ABCB1 gene encode transporter plays an important role in the first-pass elimination of orally administered drugs to limit their bioavailability by effluxing them [11,12]. The enzyme encoded by PON1 is an arylesterase that is also involved in the transformation of clopidogrel into its active state [13]. A genome-wide association analysis identified 13 SNPs on chromosome 10q24 within the CYP2C18-CYP2C19-CYP2C9-CYP2C8 cluster, showing strong evidence for association with clopidogrel response in an Amish population [14]. In a competing metabolic reaction, about 85% of the clopidogrel is hydrolyzed by CES1 to an inactive metabolite [15]. P2Y12 belongs to the G-protein coupled receptor family. It plays a key role in the ADP-dependent amplification of platelet aggregation induced by other agonists such as TBXA2 and thrombin [16,17], and it is the target of action of clopidogrel. Activation of PLA2 releases AA, which is a precursor for TBXA2 synthesis. COX1 catalyzes the first step in the formation of TBXA2 from AA. This reaction is irreversibly blocked by aspirin, which also leads to the blockage of platelet aggregation. The effects of UCP2 polymorphism on platelet reactivity and prognosis in Chinese patients with type 2 diabetes and ischemic stroke was investigated in a study, which showed that the -866G>A polymorphism was associated with clopidogrel resistance and platelet reactivity [18].

Non-responsiveness is related to recurrent ischemic events in patients with extracranial or intracranial occlusive disease on dual antiplatelet therapy, but it is not well studied. We performed this study to investigate the relationship between genetic polymorphisms and poor clinical outcomes.

## Materials and Methods

### Patient selection

This case-control study recruited consecutive ischemic stroke patients who underwent stenting for extracranial or intracranial arterial stenosis in Beijing Tiantan Hospital between May 2013 and September 2013. Patients were selected according to the following criteria: Inclusion criteria: 1) diagnosis of ischemic cerebrovascular disease with 70% to 99% stenosis of a major intracranial artery (internal carotid artery, M1 segment of middle cerebral artery, vertebral artery or basilar artery) or an extracranial artery (common carotid artery, internal carotid artery, subclavian artery, innominate artery or vertebral artery), confirmed by DSA, 2) clopidogrel (75 mg/day) plus aspirin (100 mg/day) were started at least 5 days before enrollment, 3) informed consent available. Exclusion criteria: 1) contraindications to extracranial or intracranial stenting, 2) known allergy or contraindication to aspirin, clopidogrel, heparin, local or general anesthestics, 3) active peptic ulcer disease, bleeding tendency, severe liver or kidney impairment, 4) comorbid conditions that may limit survival to less than one year, 5) enrollment in another study that would conflict with the current study. This study was approved by the Institutional Review Board of Beijing Tiantan Hospital, Capital Medical University (Ethics approval number: qx2012-012-01 and KY2014-051-01), and written informed consents were obtained from patients or their close relatives.

### Study design

Demographic and clinical characteristics of the patients, including gender, age, BMI, intracranial stent, extracranial stent, smoking, drinking, presence of hypertension, diabetes mellitus or hyperlipidemia were retrieved from the medical records. Clopidogrel 75 mg and aspirin 100 mg were given for at least 5 days before stenting. On the day before the procedure, 5 mL of venous blood from each of the patients was collected in heparin-coated tubes and stored at -70°C for genotyping.

The procedure was performed by experienced neurointerventionists, who had each done at least 100 endovascular procedures for intracranial atherosclerotic stenosis. After the procedure, dual antiplatelet therapy consisting of clopidogrel 75 mg and aspirin 100 mg were given for 90 days, followed by single antiplatelet therapy. Other medical interventions were the management of the atherosclerotic risk factors including elevated systolic blood pressure and low-density lipoprotein levels, diabetes, smoking, obesity, and insufficient exercise.

The primary endpoints were TIA, ischemic stroke, myocardial infarction and vascular-related mortality. Ischemic stroke was defined as a new focal neurologic deficit of sudden onset, lasting at least 24 hours, with no hemorrhage on CT or MRI. TIA was defined as a transient episode of neurological dysfunction caused by focal brain or retinal ischemia that lasts for at least 10 minutes but resolves within 24 hours regardless of DWI changes. The occurrence of adverse events was identified on followup visits at 1, 2, 3, 6 and 12 months, or by phone interview if the patients could not attend followups. Inpatient hospital readmission records and outpatient clinic records were reviewed. All clinical events were classified and adjudicated by two independent physicians who were blind to the follow-up process. Patients were stratified into the “case group” and the “control group”. The "control group" patients were those with no primary endpoints during a 1-year follow up. The different genotype between the two groups was compared.

### Genotyping

The most frequent genetic polymorphisms related to aspirin and clopidogrel resistance were selected. The final SNP alleles are listed in Table 1. Genomic DNA was extracted from leukocytes in the blood using the EZNA™ Blood DNA Midi Kit (Omega Bio-Tek, Norcross, GA, USA). Genotyping was performed by Boao Biotechnology Co., Ltd (Beijing, China) using the MassARRAY system (Sequenom, San Diego, CA, USA) by means of MALDI-TOF mass spectrometry method according to the manufacturer’s instructions. Single-base extension and PCR primers were designed using the Sequenom Assay Design 3.1 software (Sequenom, San Diego, CA, USA). Genotype calling was performed in real-time with the MassARRAY RT software version 3.0.0.4 and analyzed using the MassARRAY Typer software version 3.4 (Sequenom, San Diego, CA, USA). A repeat analysis of a randomly chosen subgroup of 10% of the cases and controls was conducted for quality control; the reproducibility was 100%.

**Table 1:** Selected variants for aspirin and clopidogrel.

Gene	Variant	Allele	SNP position	MAF in control	HWE *P*-value
**ABCB1**	rs3213619	A>G	5' UTR	0.044	1.000
**ABCB1**	rs1128503	A>G	Exonic, (Gly412Gly)	0.317	0.264
**ABCB1**	rs1045642	A>G	Exonic, (lle1145lle)	0.383	0.367
**CYP2C19**	rs12248560	C>T	5' UTR	0.007	1.000
**CYP2C19**	rs4244285	G>A	Exonic, (Pro227Pro)	0.319	0.313
**CYP2C19**	rs4986893	G>A	Exonic, (Trp212null)	0.046	1.000
**CYP2C19**	rs3758580	C>T	Exonic, (Val330Val)	0.103	1.000
**CYP2C9**	rs4086116	C>T	Intronic	0.107	0.141
**CYP2C18**	rs2104543	C>T	Other	0.402	1.000
**CYP2C18**	rs12772169	C>T	Other	0.395	1.000
**CYP2C18**	rs1998591	G>A	3' UTG	0.432	0.805
**CYP2C18**	rs1042194	G>T	3' UTG	0.321	0.574
**PON1**	rs662	T>C	Exonic, (Gln192Arg)	0.332	0.105
**CES1**	rs1968753	A>G	Intronic	0.398	0.365
**CES1**	rs8192950	T>G	Intronic	0.191	0.207
**P2Y12**	rs2046934	G>A	Intronic	0.181	0.121
**P2Y12**	rs6798347	A>G	Intronic	0.281	0.298
**P2Y12**	rs6801273	C>T	Intronic	0.411	0.521
**P2Y12**	rs6787801	A>G	Intronic	0.445	0.535
**COX1**	rs1330344	C>T	Promoter	0.367	0.897
**COX1**	rs10306114	C>G	5' Flanking	0.066	0.610
**UCP2**	rs659366	C>T	Promoter	0.507	0.902

*All allele are given on the positive chromosomal strand.

### Statistical analysis

Data were analyzed using SPSS statistical package version 17.0 (SPSS Inc., Chicago, Illinois, USA) and PLINK v1.07 software. Continuous variables were analyzed using the Student's *t*-test, and expressed as mean±SD. Categorical data were analyzed using Pearson's χ^2^ test, and presented as number and percentages or in the case of small expected cell frequencies, Fisher’s exact test. The HWE was tested by a χ^2^ test. The Fisher’s exact test (χ^2^ test) was used to compare the distributions of genotypes between cases and controls. Cochran-Armitage trend test was also used in our data for testing genetic association. OR and 95%CI were applied to evaluate the association of genetic variants with the primary outcome events during the follow-up period using unconditional logistic regression. Power analysis was performed using the PS Power and Sample Size Calculation program version 3.1.2. The LD pattern and haplotype structure were measured by the Haploview software 4.2 (Daly Lab, USA). The significance of any haplotypic association was evaluated using χ^2^ test. A *P*-value of less than 0.05 was considered statistically significant.

#### Clinical Trial Registration-URL

 Unique identifier: NCT01925872.

## Results

### Characteristics of enrolled population

This study enrolled a total of 268 patients including 39 cases (patients with clinical adverse events) and 229 controls (events-free patients). Their clinical characteristics are listed in Table 2. Compared with controls, patients with events had similar baseline characteristics.

**Table 2:** Clinical characteristics of patients with events and without events.

Variable	Case (n = 39)	Control (n = 229)	*P*-value
**Age, mean±SD**	62.90±7.78	62.97±9.14	0.963
**Male, n (%)**	36 (92.3)	192 (83.8)	0.170
**Intracranial stent, n (%)**	11 (28.2)	53 (23.1)	0.493
**Extracranial stent, n (%)**	28 (71.8)	176 (76.9)	0.493
**Risk factors**			
**BMI, mean±SD**	25.30±3.10	24.96±3.60	0.574
**Hypertension, n (%)**	24 (61.5)	163 (71.2)	0.226
**Diabetes, n (%)**	12 (30.8)	72 (31.4)	0.933
**Hyperlipidemia, n (%)**	12 (30.8)	89 (38.9)	0.335
**Family history of stroke, n (%)**	5 (12.8)	25 (10.9)	0.727
**Prior cerebral infarction, n (%)**	8 (20.5)	47 (20.5)	0.999
**Smoker, n(%)**			0.476
**Never**	12 (30.8)	94 (41.0)	-
**Current**	18 (46.2)	88 (38.4)	-
**Ex-smoker**	9 (23.1)	47 (20.5)	-
**Drinker, n(%)**			0.918
**Never**	20 (51.3)	125 (54.6)	-
**Social drinker**	13 (33.3)	73 (31.9)	-
**Regular drinker**	6 (15.4)	31 (13.5)	-
**Outcome, n (%)**			-
**Death**	5 (12.8)	0	-
**Ischemic stroke**	13 (33.3)	0	-
**Coronary ischemic event**	6 (15.4)	0	-
**Transient ischemic attack**	15 (38.5)	0	-

### Association of end-point and genotype

The allelic frequencies and HWE test results are shown in Table 1. Two SNPs of rs4986893 (CYP2C19*3) and rs12248560 (CYP2C19*17) had minor allele frequency (MAF) lower than 0.05. There was no significant deviation from HWE for any SNP in all the patients (*P* > 0.05). Table 3 lists the results of Fisher’s exact test and logistic regression analysis between cases and controls. Compared with controls, the cases had a significant higher mutant frequency of the allele C (PON1 rs662) (OR = 1.64, 95%CI = 1.03–2.62, *P* = 0.029), allele A (P2Y12 rs2046934) (OR = 2.01, 95%CI = 1.10–3.67, *P* = 0.041) and allele T (COX1 rs1330344) (OR = 1.85, 95%CI = 1.12–3.03, *P* = 0.017). The Cochran-Armitage trend test also suported the results of Fisher’s exact test and logistic regression analysis. The other genotypes were not significantly different between the two groups (*P* > 0.05). Statistical power was calculated to verify whether the non-significant results were really due to no relation in the sample or due to a lack of statistical power. The *P*-value of the power analysis for CYP2C19 rs4986893 was lower than 0.05, and it may be due to the the low MAF (0.046) and small sample size.

**Table 3:** Summary of SNPs detection and the results of statistical analysis.

Gene	Variant	Fisher test, *P*-value	Cochran-Armitage trend test, P-value	Logistic regression, OR, 95%CI	Power analysis
**ABCB1**	rs3213619	0.340	0.184	0.27, 0.04–2.10	0.170
**ABCB1**	rs1128503	0.191	0.177	1.39, 0.86–2.25	0.264
**ABCB1**	rs1045642	0.803	0.817	1.06, 0.66–1.71	0.057
**CYP2C19**	rs12248560	0.472	0.559	1.96, 0.20–19.30	0.162
**CYP2C19**	rs4244285	0.787	0.813	1.06, 0.64–1.78	0.057
**CYP2C19**	rs4986893	1.000	0.760	0.82, 0.23–2.90	0.047
**CYP2C19**	rs3758580	0.175	0.180	1.60, 0.80–3.20	0.299
**CYP2C9**	rs4086116	0.130	0.149	1.59, 0.84–3.01	0.298
**CYP2C18**	rs2104543	0.172	0.159	1.42, 0.87–2.31	0.301
**CYP2C18**	rs12772169	0.136	0.124	1.46, 0.90–2.38	0.341
**CYP2C18**	rs1998591	0.270	0.261	1.32, 0.81–2.15	0.207
**CYP2C18**	rs1042194	0.694	0.719	1.10, 0.66–1.82	0.068
**PON1**	rs662	0.029*	0.035*	1.64, 1.03–2.62	0.517
**CES1**	rs1968753	0.900	0.877	1.04, 0.64–1.68	0.053
**CES1**	rs8192950	0.076	0.059	0.47, 0.21–1.04	0.493
**P2Y12**	rs2046934	0.041*	0.021*	2.01, 1.10–3.67	0.710
**P2Y12**	rs6798347	0.219	0.202	1.38, 0.84–2.27	0.248
**P2Y12**	rs6801273	0.212	0.188	0.71, 0.42–1.19	0.257
**P2Y12**	rs6787801	0.903	0.885	0.97, 0.60–1.55	0.051
**COX1**	rs1330344	0.017*	0.014*	1.85, 1.12–3.03	0.709
**COX1**	rs10306114	0.804	0.660	0.78, 0.26–2.36	0.057
**UCP2**	rs659366	0.083	0.073	0.64, 0.39–1.05	0.434

*P-value < 0.05

### Haplotype analysis and association

**Fig 1:**
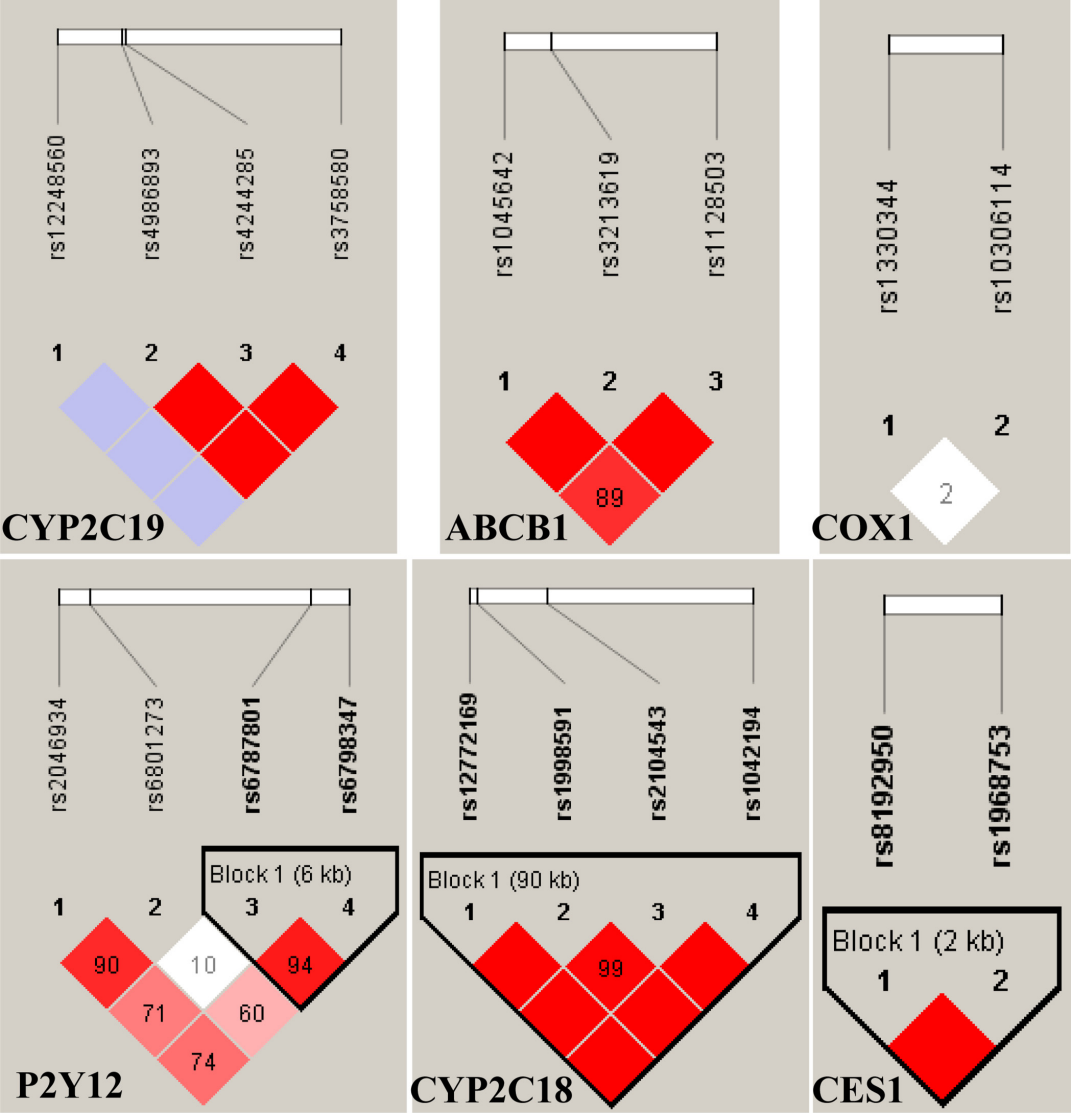
Linkage disequilibrium plot of selected SNP. SNPs in CYP2C19, ABCB1, COX1, P2Y12, CYP2C18 and CES1 genes, respectively. The depth of red color presented the computed pair-wise D'. The values in the squares are D'.

The LD block and haplotype structure were measured by D' among the selected SNPs. The final LD analysis revealed three haplotypes in our patients, and the three blocks were located in CYP2C18, CES1 and P2Y12 respectively (Fig 1. Linkage disequilibrium plot of selected SNP). The significance of any haplotypic association was shown in Table 4. Although the haplotype frequency was low (0.082), the TTTG haplotype was found to increase the risk for recurrent clinical events (*P* = 0.032). The other haplotypes may not be risk factors for recurrent clinical events for Chinese patients with extracranial or intracranial stenting.

**Table 4:** The frequency of each haplotype within a block and the association of haplotype and clinical endpoint.

Gene	Block	Haplotype Frequency	Case,Control Frequencies	*P*-value
**CYP2C18**	CCCG	0.556	0.500, 0.566	0.280
**CYP2C18**	TTTT	0.326	0.344, 0.323	0.723
**CYP2C18**	TTTG	0.082	0.144, 0.071	0.032*
**CYP2C18**	CTCG	0.034	0.013, 0.037	0.269
**CES1**	AT	0.603	0.596, 0.604	0.895
**CES1**	AC	0.217	0.290, 0.205	0.092
**CES1**	CC	0.180	0.114, 0.191	0.101
**P2Y12**	GG	0.436	0.395, 0.443	0.429
**P2Y12**	AA	0.283	0.312, 0.278	0.538
**P2Y12**	AG	0.274	0.252, 0.277	0.648

*P-value < 0.05

## Discussion

This study evaluated the impact of gene polymorphisms on adverse clinical events in patients with extracranial or intracranial occlusive disease on dual antiplatelet therapy. The results showed that PON1 rs662 (Q192R), P2Y12 rs2046934 and COX1 rs1330344 polymorphisms were significantly associated with the development of adverse clinical events.

Clopidogrel is a prodrug and is activated in the liver. Multiple enzymes, such as CYP1A2, CYP2B6, CYP2C9, CYP2C19 and CYP3A4/5 are involved in the metabolism of clopidogrel. However, the relative importance of the individual enzyme is controversial [19–21]. PON1 gene is located on the long-arm of chromosome 7, and genetic polymorphism has the biggest effect on the PON1 activity level [22]. The coding region PON1 Q192R polymorphism determines a substrate dependent effect on activity. Bouman *et al*. found that PON1 is also involved in the transformation of clopidogrel into its active state [13]. PON1 QQ192 homozygous individuals showed a considerably higher risk of stent thrombosis than RR192 homozygous individuals, lower PON1 plasma activity, lower plasma concentrations of active metabolite and lower platelet inhibition. Thus, they identified PON1 as a major determinant of clopidogrel efficacy. Several studies supported these results [23–25]. We found, in addition, that allele C of the PON1 gene was more associated with risk of clinical adverse events than allele T. However, this association was not supported by many other studies, some reporting the converse [26–29]. Other genetic and clinical factors may also influence a patient's response to clopidogrel.

P2Y12 is selectively expressed in human platelets and is a target of clopidogrel [30]. Two functional haplotypes (H1 and H2) of P2Y12 have been identified, and these haplotypes can be differentiated by any of the four tagging SNPs in absolute linkage disequilibrium. One of these SNP (rs2046934) is located in the intron [31]. This SNP showed an association with peripheral arterial disease and coronary artery disease [32,33]. However, this result was controversial, and Cuisset *et al*. found that this variant showed no influence on clopidogrel response in patients with non-ST elevation acute coronary syndrome [34]. In our patients, we found that allele A was more associated with an increased risk of clinical outcome events (TIA, ischemic stroke, myocardial infarction, and death) than allele G.

Aspirin directly and irreversibly inhibits the activity of COX-1 and COX-2 to decrease the formation of prostaglandins and thromboxane precursors from AA. The COX-1 gene contains 11 exons and most of the polymorphisms in human COX-1 gene are present at low frequency. In this study, we selected two variants with higher frequencies and analyzed their impacts on clinical outcomes. Our results showed that allele T (rs1330344) had a significant impact on adverse clinical outcome. Cao *et al*. reported that in Chinese patients with ischemic stroke treated with aspirin, TT genotype of rs1330344 might increase the risk of subsequent vascular events [35]. Another study by Fan *et al*. found that the variant T-allele of COX-1 rs1330344 was significantly associated with AR determined by LTA and thromboelastography platelet mapping assay using AA as a stimulus [36]. Similar results were also reported using AA-induced LTA combined with ADP-induced LTA to distinguish AR and non-AR [37]. However, there is no sufficient evidence on how the rs1330344 loci leads to AR. The rs1330344 polymorphism is located in the 5' UTR and may be essential for transcription. We speculate that the TT genotype may up-regulate COX-1 RNA and protein expression. Another possible reason is that rs1330344 is in linkage disequilibrium with other relevant polymorphisms which cause AR [35].

The polymorphism of the CYP2C19*3 allele (rs4986893) is a G>A transition in exon 4 that results in a premature termination codon at amino acid 212 [38]. CYP2C19*17 (rs12248560) is a C>T transition in the promoter region that creates a consensus binding site for the GATA transcription factor family, resulting in increased CYP2C19 expression and activity [39–41]. However, the CYP2C19*3 allele frequency in our patients is 4.5% and the MAF of rs12248560 is below 1%. Considering the low frequency, the two variants are unlikely significant contributors to drug non-responsiveness in our patients. CYP2C19*2 (c.681G>A, rs4244285) is a common polymorphism that results in a splicing defect and nonfunctional CYP2C19 protein [42]. CYP2C19*2 is associated with adverse cardiovascular outcomes in ACS or PCI in patients treated with clopidogrel [43–45]. However, our study did not show the same findings. The reason for these conflicting results remains unclear but may be due to several factors including a difference in study design and patient population (patients with symptomatic extracranial or intracranial occlusive disease).

In our dataset, males accounted for the majority of both cases and controls, and a separate analysis of males was performed. We got similar results: the polymorphisms of PON1 rs662 (OR = 1.86, 95%CI = 1.12–3.11, *P* = 0.016), P2Y12 rs2046934 (OR = 1.95, 95%CI = 1.10–3.47, *P* = 0.021) and COX1 rs1330344 (OR = 1.76, 95%CI = 1.06–2.92, *P* = 0.028) had significant association with recurrent clinical events. In our enrolled female patients, the data was very limited (only three cases and 37 controls), and the analytical results showed that no genetic ploymorphism was associated with the recurrent ischemic events. Due to the limited data, it was difficult to demonstrate gender differences. To investigate the impacts of other drugs (anti-hypertensive, anti-diabetic and anti-hyperlipidemic drugs) taken by patients on clinical events, logistic regression analysis was performed using SPSS software. The other drugs may not have effects on the clinical events (Table 5, *P*-value > 0.05).

**Table 5:** Multivariate logistic regression analysis of other drugs taken by the patients related to recurrent clinical events.

Variables	β*	SE^#^	Wald χ^2^	*P*-value	OR (95% CI)
**Anti-hypertensive drugs**	0.395	0.374	1.116	0.291	1.485 (0.713–3.091)
**Anti-diabetic drugs**	-0.092	0.387	0.057	0.812	0.912 (0.427–1.946)
**Anti-hyperlipidemic drugs**	0.294	0.382	0.594	0.441	1.342 (0.635–2.838)
**Constant**	-2.031	0.386	27.716	0.000	0.131

*β: regression coefficient

^#^SE: standard error of regression coefficient.

Based on the pathway of aspirin and clopidogrel metabolism, PON1 and P2Y12 are the clopidogrel-metabolizing enzyme and target, and COX1 is the pharmacological target of aspirin. According to genetic testing, several therapeutic options may be selected. Firstly, an increase in the dose of aspirin or clopidogrel might reduce the rate of poor response. Secondly, aspirin or clopidgrel may be switched to other antiplatelet drugs.

Several limitations of this study need to be mentioned: 1) the sample size is small with only 268 patients (39 cases) meeting the inclusion and exclusion criteria, 2) not all platelet function tests were performed in the current study, and we will analyze the association of genetic variants and platelet function in future study, and 3) P2Y12 and COX1 gene expression had not been analyzed.

## Conclusions

In summary, in Chinese patients with extracranial or intracranial occlusive disease, PON1 rs662, P2Y12 rs2046934 and COX1 rs1330344 genetic polymorphisms may increase the risk of subsequent vascular events. None of the other previously reported SNPs with suggested pharmacogenetic influence on antiplatelet therapy efficacy were associated with outcome events during 1 year follow-up in our study. However, this is a relatively small-scale study and the results still need to be verified by a larger study.

## Abbreviations

AA: arachidonic acid
ABCB1: ATP-binding cassette sub-family B member 1
ACS: acute coronary syndromes
ADP: adenosine diphosphate
AR: aspirin resistance
BMI: body mass index
CES1: carboxylesterase 1
CI: confidence interval
COX-1: cyclooxygenase-1
COX-2: cyclooxygenase-2
CYP1A2: cytochrome P450 family 1 subfamily A polypeptide 2
CYP2B6: cytochrome P450 family 2 subfamily B polypeptide 6
CYP2C8: cytochrome P450 family 2 subfamily C polypeptide 8
CYP2C9: cytochrome P450 family 2 subfamily C polypeptide 9
CYP2C18: cytochrome P450 family 2 subfamily C polypeptide 18
CYP2C19: cytochrome P450 family 2 subfamily C polypeptide 19
CYP3A4: cytochrome P450 family 3 subfamily A polypeptide 4
CYP3A5: cytochrome P450 family 3 subfamily A polypeptide 5
DSA: digital subtraction angiography
DWI: diffusion weighted imaging
HWE: Hardy-Weinberg equilibrium
LD: linkage disequilibrium
LTA: light transmission aggregometry
MAF: minor allele frequency
MALDI-TOF: matrix-assisted laser desorption ionization time-of-flight
OR: odds ratios
P2Y12: purinergic receptor P2Y, G-protein coupled, 12
PCI: percutaneous coronary intervention
PLA2: phospholipase A2
PON1: paraoxonase 1
SD: standard deviation
SE: standard error
SNPs: single nucleotide polymorphisms
TBXA2: thromboxane A2
TIA: transient ischemic attack
UCP: uncoupling protein 2
UTR: untranslated region
